# The Differential Proliferative Ability of Satellite Cells in Lantang and Landrace Pigs

**DOI:** 10.1371/journal.pone.0032537

**Published:** 2012-03-13

**Authors:** Xiu-qi Wang, Wei-jun Yang, Zhou Yang, Gang Shu, Song-bo Wang, Qing-yan Jiang, Li Yuan, Tong-shan Wu

**Affiliations:** 1 College of Animal Science, South China Agricultural University, Guangzhou, Guangdong Province, China; 2 Guangdong Provincial Key Laboratory of Agro-Animal Genomics and Molecular Breeding, Guangzhou, China; 3 College of Life Science, Xiamen University, Xiamen, Fujian Province, China; 4 Swine Breeding Center of Banling, Shaoguan, Guangdong Province, China; Laboratoire Arago, France

## Abstract

Here, for the first time, we evaluate the hypothesis that the proliferative abilities of satellite cells (SCs) isolated from Lantang (indigenous Chinese pigs) and Landrace pigs, which differ in muscle characteristics, are different. SCs were isolated from the longissimus dorsi muscle of neonatal Lantang and Landrace pigs. Proliferative ability was estimated by the count and proliferative activity of viable cells using a hemocytometer and MTT assay at different time points after seeding, respectively. Cell cycle information was detected by flow cytometry. Results showed that there was a greater (*P*<0.05) number of SCs in Lantang pigs compared with Landrace pigs after 72 h of culture. The percentage of cell population in S phase and G_2_/M phases in Lantang pigs were higher (*P*<0.05), while in G_0_/G_1_ phase was lower (*P*<0.05) in comparison with the Landrace pigs. The mRNA abundances of MyoD, Myf5, myogenin and Pax7 in SCs from Lantang pigs were higher (*P*<0.05), while those of myostatin, Smad3 and genes in the mammalian target of rapamycin (mTOR) pathway (with the exception of 4EBP1) were lower (*P*<0.05) than the Landrace pigs. Protein levels of MyoD, myogenin, myostatin, S6K, phosphorylated mTOR and phosphorylated eIF4E were consistent with the corresponding mRNA abundance. Collectively, these findings suggested that SCs in the two breeds present different proliferative abilities, and the proliferative potential of SCs in Lantang pigs is higher than in Landrace pigs.

## Introduction

Different breeds and lines have a predetermined propensity towards excellence in certain areas, such as carcass composition and meat quality [1], [2]. The Langtang pig is native to the southern of China and is characterized by earlier sexual maturity and lower growth performance as well as by generally possessing more intramuscular fat and better meat quality than most commercial European pigs, such as Landrace pigs [3]. During postnatal skeletal muscle growth, fiber hypertrophy is accompanied by the proliferation of satellite cells (SCs), which are the source of new nuclei incorporated into muscle fibers. These developmental processes are primarily controlled by a group of transcriptional networks, known as myogenic regulatory factors (MRFs), which includes MyoD, Myf5, MRF4 and myogenin [4], [5]. Pax7, the paired box transcription factor expressed nearly ubiquitously by quiescent SCs, is co-expressed with MyoD in proliferating myoblast progeny [6]. Pax7 directs the induction of target genes such as Myf5, which regulates SC entry into the myogenic program [7], [8]. Coordinated increases in both cell number and cell size contribute to the growth of an organ or whole organism [9]. Myostatin, also known as growth differentiation factor 8, prevents myoblast progression from the G_1_ phase to S phase of the cell cycle [10]. The mammalian target of rapamycin (mTOR) pathway, regulated by its upstream targets Akt and TSC1/2, is one of the major mechanisms for protein synthesis regulation [11], [12], and controls mammalian cell size and cell cycle progression via its downstream targets p70S6K and eIF4E [13], [14].

During the postnatal life of pig, muscle growth is dependent on SC proliferation, differentiation and fusion to increase the DNA content of existing muscle fibers and their capacity for protein synthesis. Thus, primary muscle SCs can serve as an excellent *in vitro* system for evaluating the various stages of muscle development. Little is known about the proliferative potential of SCs isolated from different pig breeds. Thus, this study was conducted with SCs isolated from Lantang and Landrace pigs to test the hypothesis that muscle fibers from different pig breeds have different proliferative abilities during the neonatal stage.

## Methods

### Sampling

Three Lantang or Landrace male pigs at 1 day of age were obtained, respectively, from the Experimental Center for Swine Breeding of South China Agricultural University, Guangdong Province, China. The pigs were sacrificed by lethal injection of sodium pentobarbital. Three kinds of muscle tissues (longissimus dorsi, LD; semitendinosus, ST; semimembranosus, SM) were sampled. Each individual sample was separated and frozen immediately in liquid nitrogen and stored at −80°C for muscle frozen section and morphologic analysis. All procedures were approved by the Animal Care Committee of South China Agricultural University (Guangzhou).

### Morphologic analysis of muscles

The muscle samples were cross-sectioned at 10 µm using a Leica RM2235 (German) and placed on positively charged glass slides for histological examination. The slices were stained with hematoxylin-eosin. All samples were examined with an image processing system (Motic China Group Co., Ltd). The system consisted of an optical microscope and a standard workstation computer that controlled the image analysis. Fiber number and cross section area (CSA) were obtained from the slices, and cell number counts were performed across five separate fields of view for at least three slices of each muscle sample.

### Isolation, purification and characterization of satellite cells

SCs from Lantang or Landrace pigs were isolated from the longissimus dorsi muscle as described by Doumit [15] and Singh [16], with some changes. Briefly, the isolated muscle tissue was washed with DMEM/F12 medium (Gibco, Grand Island, NY) and excised, trimmed of visible connective tissue, and minced with fine sharp scissors in dishes. Minced muscle was treated for 60 min with 0.2% collagenase (Collagenase Type II, Sigma, St. Louis, MO) in a 37°C water bath. This was followed by centrifugation at 1,500×g and 4°C for 10 min. The pellet was washed with DMEM/F12 medium and centrifuged 3 times at 800×g and 4°C for 10 min and then passed through a 200-mm filter. Subsequently, a 50-mm filter was utilized to separate mononuclear cells from the muscle fibers and myofibril fragments. The resulting supernatants were centrifuged at 800×g for 5 min. At this point, the supernatant fluid was discarded. The cells were pre-plated repeatedly to remove fibroblasts, as described previously [17]. The muscle SCs were plated in a growth medium (GM) containing 90% DMEM/F12, 10% fetal bovine serum (FBS) (Gibco, Grand Island, NY), 15 mmol/mL HEPES, 100 U/mL penicillin-streptomycin and 2 mmol L-glutamine (all reagents were obtained from Invitrogen, Carlsbad, CA). The cells were incubated at 37°C and 5% CO_2_ in a standard cell culture incubator (Shellab, USA).

### Identification of satellite cells

SCs isolated from Lantang or Landrace pigs were seeded at a density of 1×10^3^ cells/well into 96-well plates for immunocytochemical analysis and 1×10^5^ cells/well into 6-well plates for differentiation analysis. For immunocytochemical analysis, the cells were fixed with 80% cool acetone for 20 min at room temperature and washed in Ca^2+^- and Mg^2+^-free phosphate-buffered saline (PBS) 3 times. One hundred microliters of 3% H_2_O_2_ diluted with distilled water was added to the cells for 5 min at room temperature to deactivate endogenous enzymes. After blocking with 100 µL of 5% bovine serum albumin, a mouse monoclonal antibody against desmin (1∶100, Boster Bio-engineerting Co. Ltd., Wuhan, China) was added as primary antibody and then incubated at 37°C for 1 h. A negative control was performed by replacing the primary antibody with buffer. After washing 3 times with PBS, the cells were stained with a SABC kit (Boster Bio-engineerting Co. Ltd., Wuhan, China) followed by DAB staining using a Histostain-Plus kit (Jingmei Biotech Co. Ltd., Shenzhen, China).

For differentiation analysis, the medium was changed to myogenic differentiation medium as modified from a previous report [18], which was DMEM/F12 containing 2% horse serum (Invitrogen, Carlsbad, CA) and 100 U/mL penicillin-streptomycin. The hematoxylin-eosin staining procedure was used to determine the differentiation of the SCs between Lantang and Landrace pigs. For the hematoxylin-eosin staining, the cells were rinsed twice with Ca^2+^- and Mg^2+^-free PBS followed by fixation in 4% paraformaldehyde in PBS (w/v) for 30 min at room temperature. The cells were stained with hematoxylin-eosin and photographed.

### Proliferation activity analysis

#### The cell count assay

The cells were cultured in a 6-well plate at a density of 5×10^4^ cells/mL in GM and collected using Trypan Blue, as previously described [19]. Viable cells were counted using a hemocytometer under a light microscope after cultured at 24, 48, 72, 96, 120 and 144 h, respectively. Three replicates were performed, and the cells were isolated from a different piglet for each replicate.

#### The MTT assay

The cells were cultured in a 96-well plate at a density of 2×10^3^ cells/mL in GM, as previously described [20], [21], with minor changes. Twenty microliters of MTT (5 mg/mL, Sigma, St Louis, MO) solution were added to each well and incubated for 4 h. The microtiter plates were centrifuged at 1,400×g and 4°C for 5 min, and the untransformed MTT solution was carefully removed using an Eppendorf pipette. To each well, 200 µL of DMSO working solution (180 µL DMSO with 20 µL 1 mol/L HCl) was added, and the OD of the yellow reaction product was evaluated in an ELISA reader at a 490-nm wavelength. At least 3 independent experiments from different piglets were performed to examine SC proliferation.

#### Flow cytometry

The cells were cultured in a 6-well plate at a density of 1×10^5^ cells/mL in GM and collected at 48, 72, 96 and 120 h, respectively, as previously described [22] with minor changes. Briefly, the cells were fixed with 4 mL cold ethanol and carefully stored at −20°C in fixation buffer until the samples were ready for analysis. After that, the fixed cells were carefully centrifuged at 200×g and 4°C for 10 min, resuspended in 1 mL PBS, treated with 100 µL of 200 µg/mL DNase-free, RNaseA and incubated at 37°C for 30 min. Finally, the cells were treated with 100 µL of 1 mg/mL propidium iodide (light sensitive), incubated at room temperature for 5–10 min and subjected to flow cytometry using a Becton Dickinson FACScan (BD, USA).

### Cell size analysis

The SCs from Lantang or Landrace pigs were seeded into 96-well plates at a density of 1×10^3^ cells/well to examine the cell size. For hematoxylin-eosin staining, cells were harvested at 72 h and rinsed twice with Ca^2+^- and Mg^2+^-free PBS followed by fixation in 4% paraformaldehyde in PBS (w/v) for 30 min at room temperature. The cells were stained with hematoxylin-eosin and photographed. The pictures were examined using an image processing system (NIS-Elements, Nikon, Japan).

### RNA isolation and Reverse transcription (RT)

Total RNA was isolated from the cultured cells at 72 h using Trizol reagent (Invitrogen, Carlsbad, CA), by following the instructions of the manufacturer, and RNA quality was assessed by agarose gel electrophoresis (1%). The RNA had an OD260∶OD280 ratio between 1.8 and 2.0. Reverse transcription was performed using 1 µg of total RNA and Moloney murine leukemia virus reverse transcriptase (Invitrogen, Carlsbad, CA). The reverse transcription conditions for the cDNA amplification were 65°C for 5 min, 37°C for 30 min, and 70°C for 15 min. Synthesis of the cDNA first strand was performed with random primers (N10) and Superscript II reverse transcriptase (Invitrogen, Carlsbad, CA).

### Quantitative real-time polymerase chain reaction (qRT-PCR)

qRT-PCR was performed using one-step SYBR Green PCR Mix (Takara, Dalian, China) containing MgCl_2_, dNTPs, and Hotstar Taq polymerase. Primers were designed specifically for each gene using Primer 5.0 software. Amplification and melting curve analysis were performed in a Stratagene Mx3005P real-time PCR system (Stratagene, USA). Melting curve analysis was conducted to confirm the specificity of each product, and the sizes of products were verified on ethidium bromide-stained 1.0% agarose gels in Tris acetate-EDTA buffer. The relative mRNA expression was calculated by 2^−ΔCt^ (ΔCt = ΔCt of the target gene-ΔCt of the housekeeping gene) [23]. Real-time PCR analysis of each sample was repeated three times. Moreover, to generate gene-specific standard curves, each gene's cDNA product was serially diluted from 10^−1^ to 10^−8^ and used as the PCR template to detect the amplification efficiency of each primer set. Primers and standard curve data (R^2^, slope, and efficiency) were given in Table 1.

**Table 1:** Primer sequences and standard curve data for quantitative real-time PCR analysis.

Gene	Primer sequence	Product size (bp)	R^2^ A	Slope^B^	Efficiency (%)^C^
	F:5′-GCCTGTCCGCAGAAGATGG-3′				
Myf5	R:5′-CGTGGCTCAAACTCGTCCC-3′	119	0.999	−3.294	101.2
	F:5′-TCTATGATGACCCGTGTTT-3′				
MyoD	R:5′-CAGGGAAGTGCGAGTGTT -3′	114	0.999	−3.332	99.6
	F:5′-CCAGGAACCCCACTTCTATG-3′				
Myogenin	R:5′-GTCCCCAGCCCCTTATCTT-3′	138	0.999	−3.389	97.2
	F:5′-GATTATCACGCTACGACGGA-3′				
Myostatin	R:5′-GAAGCAGCATTTGGGTTTT-3′	90	0.999	−3.256	102.8
	F:5′-CCCTGTGTCATCTCCCGC-3′				
Pax7	R:5′-CCACCTGTCTGGGCTTGC-3′	124	0.999	−3.295	101.1
	F:5′- AGGAGAAGTGGTGCGAGAA -3′				
Smad3	R:5′- ACAGGCGGCAGTAGATGAC -3′	195	0.999	−3.264	102.5
	F:5′-ACAACCAGGACCACGAGAAG -3′				
Akt	R:5′-GAAACGGTGCTGCATGATCT -3′	173	0.999	−3.286	101.5
	F:5′-GGAACTGGAATCTCATCTGG -3′				
TSC1	R:5′-TCTTCTGAGCCTCGTACCTG -3′	111	1.000	−3.422	96.0
	F:5′-GGTTTGATTATGGTCACTGG -3′				
mTOR	R:5′-TGCAATGAGCTGAGGTATAA -3′	104	0.998	−3.317	100.2
	F:5′- AGTGTCGGAACTCACCTGT-3′				
4EBP1	R:5′-TTCTGGCTGGCATCTGT -3′	106	0.999	−3.105	109.9
	F:5′-TACTGTTGAAGACTTTTGGG -3′				
eIF4E	R:5′-CACATAGGCTCAATACCATC -3′	105	0.997	−3.135	108.4
	F:5′-TGGAACTCTTAGGCACATCA -3′				
S6K	R:5′-CGACACAATCAGAACTCAAC -3′	154	0.987	−3.121	109.1
	F:5′-ATTCAGCGTCTTGTTACTCC -3′				
RPS6	R:5′-CTCCGTCTCTTGGCAATCT -3′	173	0.999	−3.369	98.1
	F:5′-GGTCGGAGTGAACGGATTTG-3′				
GAPDH	R:5′-CCTTGACTGTGCCGTGGAAT -3′	170	0.999	−3.391	99.2

A : Regression coefficient of the standard curve;

B : The slope of the standard curve;

C : The amplification efficiency of the PCR. As detailed in introduction, MyoD, Myf5, MRF4 and myogenin are examples of MRFs. Akt and TSC1/2 are upstream targets of mTOR while 4EBP1, eIF4E, S6K and RPS6 are downstream targets. Pax7 and myostatin also control myoblast proliferation.

### Western blot assay

The cells were homogenized using lysis buffer [50 mmol/mL Tris-HCl, pH 7.5, 150 mmol/mL NaCl, 1 mmol/mL EDTA, 0.5% Triton X-100, 1 mmol/mL phenylmethanesulfonyl fluoride (PMSF), 1 mmol/mL Na_3_VO_4_] containing complete protease inhibitor (Invitrogen, Carlsbad, CA). Homogenates were centrifuged at 14,000×g and 4°C for 15 min, and the protein concentration in the supernatants was determined using a BCA Protein Assay Reagent Kit (Pierce, Rockford, IL). Protein samples were boiled for 5 min and then subjected to 15% SDS gel electrophoresis. The proteins were separated by electrophoresis at 80 V for 20 min and 120 V for 100 min using Tris-glycine running buffer (0.025 mol/L Tris base, 0.192 mol/L glycine, and 0.1% SDS, pH 8.3). Prestained molecular weight markers (Invitrogen, Carlsbad, CA) were used to determine the molecular weight of proteins. The proteins were subsequently electro-transferred onto polyvinylidene difluoride (PVDF) membranes using transfer buffer that contained 25 mmol/L Tris base, 192 mmol/L glycine, and 10% methanol, pH 8.1 to 8.3.

Membranes were blocked for 1 h in 5% BSA and TBS buffer (20 mmol/L Tris and 500 mmol/L NaCl, pH 7.6) at room temperature. The membranes were incubated overnight at 4°C with 1∶5000 rabbit monoclonal antibody against eIF4E phospho(pS209) (Epitomics, Inc., California), 1∶250 rabbit polyclonal antibody against S6K (Abcam, Cambridge, UK), 1∶1000 rabbit monoclonal antibody against mTOR/FRAP phospho(pS2448) (Epitomics, Inc., California, US), 1∶600 mouse polyclonal antibody against myostatin (Santa Cruz Biotechnology, Santa Cruz, CA), 1∶800 mouse polyclonal antibody against MyoD (Santa Cruz Biotechnology, Santa Cruz, CA), 1∶800 goat polyclonal antibody against myogenin (Santa Cruz Biotechnology, Santa Cruz, CA), and 1∶1000 mouse monoclonal antibody against β-actin (Santa Cruz Biotechnology, Santa Cruz, CA). All dilutions were made in TBST buffer (TBS buffer with 0.05% Tween-20). Membranes were washed 3 times for 10 min with TBST buffer followed by incubation for 1 h with horseradish peroxidase-labeled anti-mouse, anti-goat and anti-rabbit IgG (Santa Cruz Biotechnology, Santa Cruz, CA). The proteins were visualized with the ECL-Plus western blotting reagent (Amersham Pharmacia Biotech, Piscataway, NJ) and exposed to Kodak X-Omat film. The density of bands was analyzed using Image Analysis Software (Tanon, China).

### Statistical analysis

Data are expressed as the mean ± SEM. Results were analyzed with Student's t test to determine any significant differences between two groups by SAS (SAS Institute, 2001) [24]. *P*<0.05 indicates significance.

## Results

### The morphology of the muscle tissues

**Figure 1:**
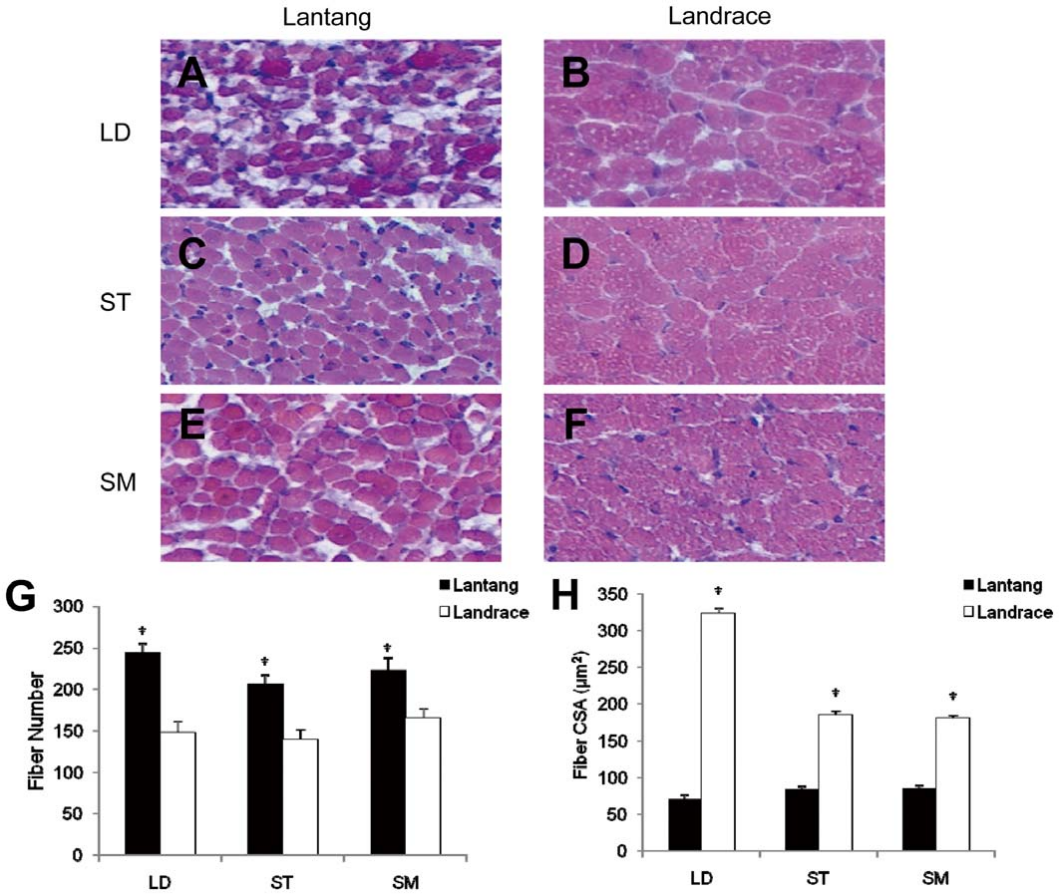
Fiber number and cross-sectional area differences in three muscle tissues in Lantang and Landrace pigs. CSA: cross-sectional area; LD: longissimus dorsi; ST: semitendinosus; SM: semimembranosus. LD, ST and SM muscles in Lantang (A, C and E) pigs and Landrace pigs (B, D and F) were stained with hematoxylin-eosin for histological examination. Quantification of fiber number and CSA of LD, ST and SM muscles in Lantang pigs and Landrace pigs (G–H). The original magnification was 400×. The results are representative of 3 separate experiments. Bars are means ± SEM. * Indicates a significant difference (*P*<0.05).

Fiber number and cross-sectional area (CSA) differences in the three muscle tissues (LD, ST and SM muscle) in Lantang and Landrace pigs were examined using the muscle frozen sections. Histologic examination of the hematoxylin- and eosin-stained cross sections of LD muscle revealed that Lantang pigs show a significantly greater (1.7-fold) (*P*<0.05) fiber number and significantly lower (4.6-fold) (*P*<0.05) CSA in comparison with the Landrace pigs (Fig. 1 A & B, G & H). In ST muscle, the fiber number in Lantang pigs was significantly greater (1.5-fold) (*P*<0.05) than in the Landrace pigs, and Landrace pigs showed significantly greater (2.2-fold) (*P*<0.05) CSA compared with Lantang pigs (Fig. 1 C & D, G & H). The same results were found in SM muscles (Fig. 1 E & F, G & H). Collectively, these findings showed that the fiber number and CSA between Lantang and Landrace pigs were different. Based on this observation, we hypothesized that SC proliferation in Lantang and Landrace pigs is also different.

### Identification of SCs

**Figure 2:**
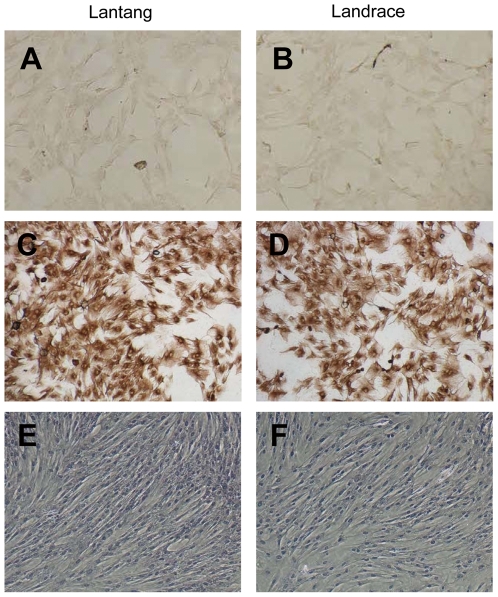
Identification of satellite cells (SCs) by immunocytochemistry and hematoxylin–eosin staining. SCs from Lantang and Landrace pigs were seeded in 96- and 6-well plates for immunocytochemistry (A–D) and hematoxylin–eosin staining (E and F). (C) and (D) represent SCs immunolabelled with mouse monoclonal anti-desmin antibodies. In the negative control (A and B), this primary antibody was withdrawn (The original magnification was 100×). (E) and (F) represent SCs in Lantang and Landrace pigs stained with hematoxylin–eosin. The staining is three days after differentiating (The original magnification was 100×).

Immunocytochemistry analysis showed that the isolated SCs were positive for the mouse monoclonal antibody against desmin (Fig. 2 A–D). The cells were induced to become myotubes using 2% horse serum, and stained with hematoxylin-eosin after 3 days of differentiation (Fig. 2 E & F).

### The difference in proliferation potential in SCs

**Figure 3:**
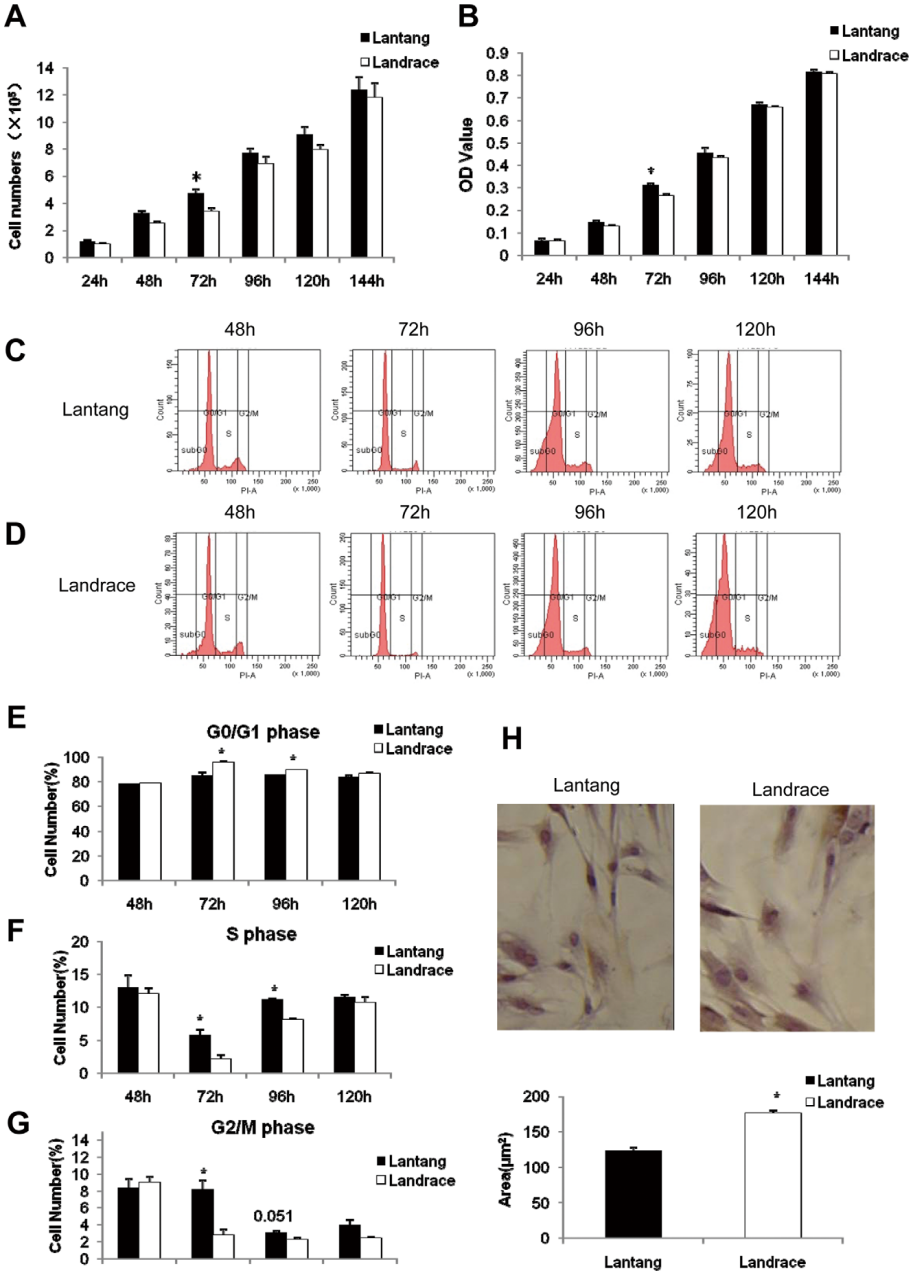
Proliferation of satellite cells (SCs) in Lantang and Landrace pigs. SCs were cultured in DMEM/F12 medium supplemented with 10% FBS. Cell number was tested using cell counts (A) and the MTT assay (B) at 24 h, 48 h, 72 h, 96 h, 120 h and 144 h. The cell cycle was examined by flow cytometry at 48 h, 72 h, 96 h and 120 h (C and D). Representative frequency curve of the cell-cycle profile obtained from analyzing the cells which cultured at 48 h, 72 h, 96 h and 120 h. Cell cycle was determined by counting all the cells in the sample and plotting their respective DNA content. The two prominent peaks represent G0/G1 and G2/M phase cells, respectively. The intermediate region between peaks represents S phase cells (C and D). Statistical analysis of the percentage of cell number in G0/G1, S and G2/M phase at the four time points is reported in (E–G). The cell size of SCs in Lantang and Landrace pigs (H) (The original magnification was 200×). The results are representative of 3 separate experiments. Bars are means ± SEM. * Indicates a significant difference (*P*<0.05).

Based on morphologic analysis, differences in the number of viable cells were tested by cell count analysis and MTT assay. Our results showed that when the different time points were analyzed separately, no significant differences in the number of viable cells were found from 24 to 144 h, whereas at 72 h, a significantly greater number of viable cells (1.4-fold for the cell count analysis, 1.2-fold for the MTT assay) (*P*<0.05) was found in Lantang pigs than in Landrace pigs. Furthermore, the SC number in Lantang pigs was higher (*P*>0.05) than in Landrace pigs at 24, 48, 96, 120 and 144 h (Fig. 3 A & B). Even though there was no overall significant difference in the proliferation of SCs between Lantang and Landrace pigs, the number of viable cells in Lantang pigs increased faster than in Landrace pigs after 48 h. To better understand the *in vitro* SC proliferation, the cells cultured at 48, 72, 96 and 120 h were tested by flow cytometry, respecyively. DNA content distribution of cells obtained from Lantang pigs and Landrace pigs consisted of two predominant peaks corresponding to cells in G0/G1 and G2/M cell cycle phases, respectively. The intermediate region between both peaks included cells in the S phase of cell cycle (Fig. 3 C & D). The statistical results showed that the percentage of cell population in S and G_2_/M phases in Lantang pigs were significantly greater (2.65 and 2.86-fold, respectively) (*P*<0.05), whereas in G_0_/G_1_ phase, it was significantly lower (1.1-fold) (*P*<0.05) compared with Landrace pigs at 72 h. At 96 h, the percentage of cell number in S phase in Lantang pigs was significantly greater (1.38-fold) (*P*<0.05) than in Landrace pigs while in G_0_/G_1_ phase significantly lower (1.1-fold) (*P*<0.05) compared with Landrace pigs. No significant differences were found at 48 and 120 h (Fig. 3 E–G). Consistent with the findings of the cell count analysis and MTT assay, our results suggested that SC proliferation in Lantang pigs was different from Landrace pigs. Meanwhile, to determine the cell size at proliferation of 72 h, SCs were seeded at a density of 1.0×10^3^ cells/well in 96-well plates for hematoxylin-eosin staining. Results indicated that the SC size in Landrace pigs was significantly larger (1.43-fold) (*P*<0.05) than in Lantang pigs (Fig. 3 H). These findings were consistent with the morphologic analysis.

### Gene expression levels and protein amounts in SCs after 72 h proliferation

**Figure 4:**
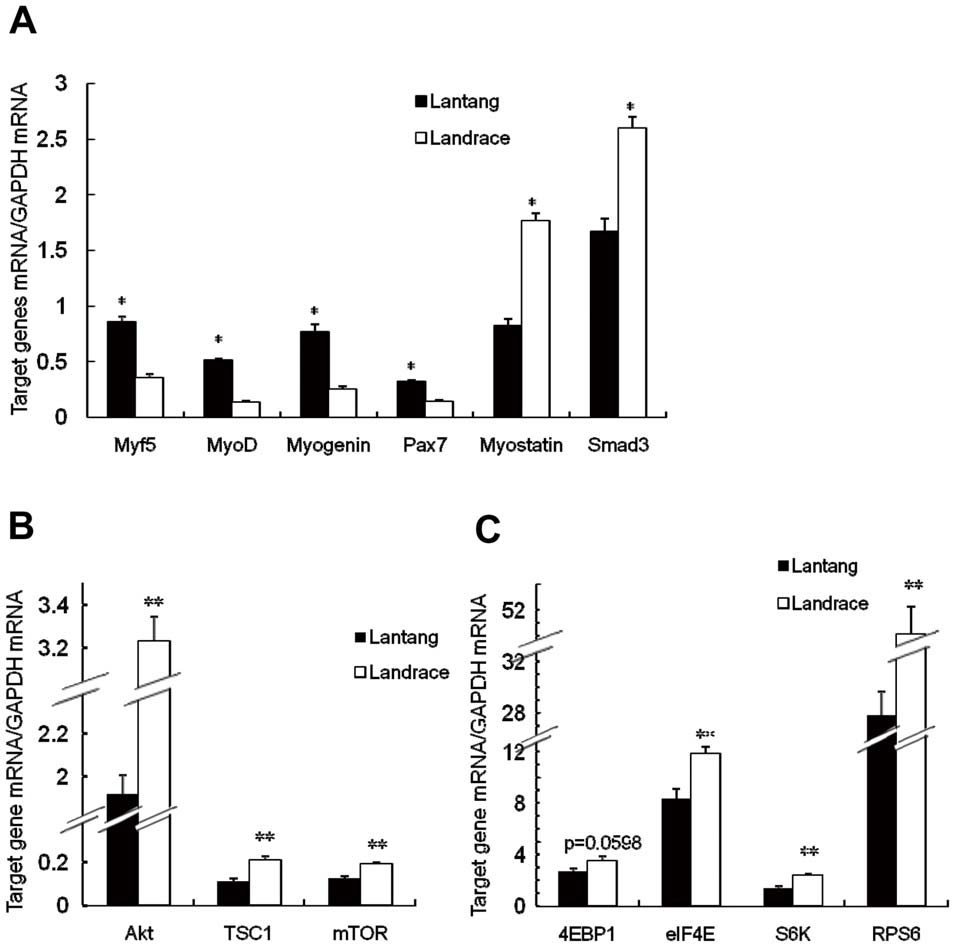
Gene expression levels of Pax7, Myostatin, Smad3, myogenic regulatory factors and mammalian TOR (mTOR) in satellite cells (SCs) in Lantang and Landrace pigs during 72 h proliferation. Relative levels of Pax7, Myostatin, Smad3, myogenic regulatory factors (A) and mammalian TOR (mTOR) (Band C) were measured by quantitative real-time PCR. Glyceraldehyde-3-phosphate dehydrogenase (GAPDH) was used as a housekeeping gene. The results are representative of 3 separate experiments. Bars are the mean ± SEM. * and **Indicate a significant difference (*P*<0.05) and (*P*<0.01).

Along with the differences in the morphology and SC proliferation potential between Lantang and Landrace pigs, the mRNAs levels of the MyoD, Myf5, myogenin, Pax7, myostatin, Smad3 and key genes in mTOR pathway were tested. The results showed that the mRNA levels of MyoD, Myf5, myogenin and Pax7 in SCs in Lantang pigs were significantly higher (3.1, 2.4, 3.5 and 2.2-fold, respectively) (*P*<0.05) than in Landrace pigs. However, the mRNA levels of myostatin and Smad3 in SCs in Landrace pigs were significantly higher (2.1 and 1.6-fold, respectively) (*P*<0.05) than in the Lantang pigs (Fig. 4 A). Interestingly, when the transcript levels of key genes in the mTOR pathway were detected, it was found that gene expression in the SCs in Landrace pigs was markedly higher (*P*<0.01) (except for 4EBP1, *P* = 0.0598) than in Lantang pigs (Fig. 4 B & C).

**Figure 5:**
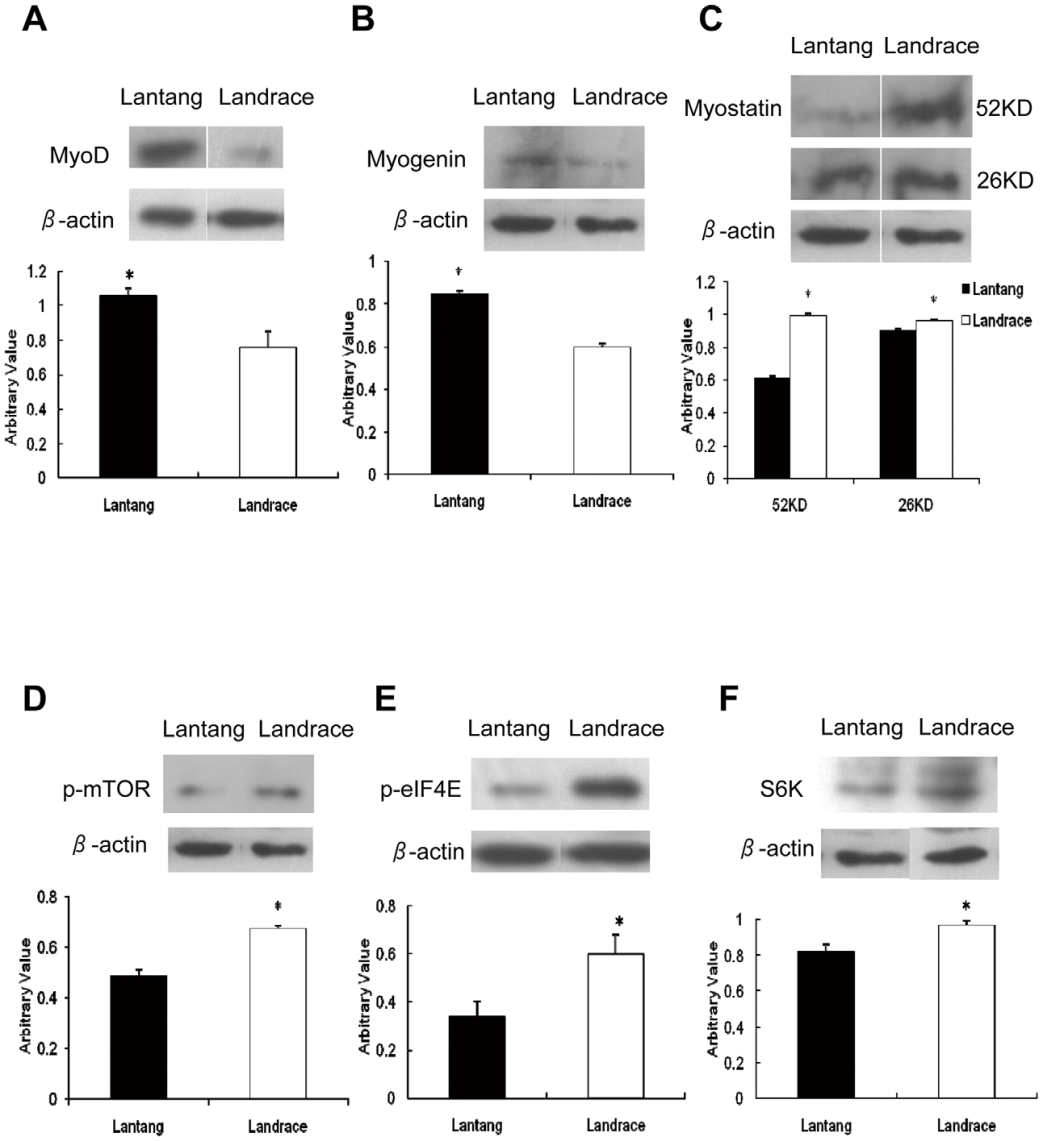
Western blot analysis of satellite cells for MyoD, Myogenin, Myostatin, p-mTOR, p-eIF4E and S6K during proliferation at 72 h. Electrophoresed protein was transferred to a polyvinylidene difluoride membrane by electrophoretic blotting. The membrane was incubated with primary antibodies for (A) MyoD, (B) Myogenin, (C) Myostatin, (D) p-mTOR, (E) p-eIF4E, (F) S6K with horseradish peroxidase- labeled anti-mouse, anti-goat and anti-rabbit IgG. Proteins were visualized with the ECL-Plus Western Blotting Reagent (Amersham Pharmacia Biotech, Piscataway, NJ) and exposed to Kodak X-Omat film. The density of the bands was analyzed using Image Analysis Software (Tanon, China). The results are representative of 3 separate experiments. Bars are the mean ± SEM. * Indicates a significant difference (*P*<0.05).

Antibodies to the cellular proteins were also used for western blot analysis of porcine SC proteins at 72 h proliferation. Our results revealed that the protein levels of MyoD and Myogenin in SCs in Lantang pigs were significantly higher (1.38 and 1.41-fold, respectively) (*P*<0.05) than in Landrace pigs (Fig. 5 A & B). Myostatin protein was detected in its precursor form (52 kDa) as well as the mature peptide (26 kDa) in all samples. The protein levels of both forms of Myostatin were significantly higher (1.6 and 1.1-fold, respectively) (*P*<0.05) in the SCs from Landrace pigs than in Lantang pigs (Fig. 5 C). Furthermore, phosphorylated mTOR, phosphorylated eIF4E and S6K protein levels were significantly higher (1.66-, 1.74 and 1.18-fold, respectively) (*P*<0.05) in SCs from Landrace pigs than in Lantang pigs (Fig. 5 D–F).

## Discussion

In this study, Lantang and Landrace pigs were selected as neonates to validate the hypothesis that there was difference in the proliferative ability of SCs between different breed pigs during neonatal period.

During postnatal growth, the increase in skeletal muscle mass is mainly due to increased muscle fiber number and size. Postnatal fiber hypertrophy, which is associated with the accumulation of myonuclei (SC proliferation) and muscle-specific proteins, is correlated with the number of prenatally-formed muscle fibers. Furthermore, the number is fixed before birth, and fiber formation ceases at approximately 85–90 days of gestation. This cessation corresponds to when the total number of fibers is established in pigs [25]–[28]. Thus, the total number of muscle fibers is an important aspect of postnatal muscle growth. In this study, three muscle tissues (LD, ST and SM muscle) were used to determine the differences in fiber number and CSA in Langtang and Landrace pigs. Based on morphologic analyses, the fiber numbers in the three muscle tissues from Lantang pigs were significantly higher, while the CSA was significantly lower than from Landrace pigs (Fig. 1 A–G). There are a few studies comparing muscle fiber number and CSA between different pig breeds. Staun [29] showed that fiber number in LD muscle in Piétrain pigs was lower and CSA was higher than in Danish Landrace pigs. The same results were found when muscle fiber number and CSA were compared between two different animals [30]. In that report, the pig LD muscle shows a higher fiber number and lower CSA compared with the extensor digitorum longus muscle of the mouse. Our data are similar to studies by Staun [29] and Rehfeldt [30]. Previous studies indicated that muscle fiber number and muscle cross-sectional area had a profound influence on meat quality traits. In pigs, muscle fiber size and capillary density seem to be important factors that influence the meat quality and metabolic response at time of slaughter [31]. Our findings suggested that differences between Langtang and Landrace pigs in fiber number and CSA may relate to the meat quality. The SCs in these two pig breeds may be the difference in proliferative potential.

To test the difference in SC proliferation in the two breeds, an *in vitro* porcine primary muscle SC culture system was used. In this study, the SCs were isolated from the LD muscle from Lantang and Landrace pigs. In this system, proliferative ability was measured as the number of viable cells over a short period of 6 days. Although not significant for all time points, a tendency towards an overall difference in SC proliferation in Lantang and Landrace pigs was found. Specifically, the number of viable cells was significantly higher in Lantang pigs than in Landrace pigs at 72 h (Fig. 3 A & B), implying that the SC proliferation rate was slower in Landrace pigs. Similar results were observed for muscle SCs isolated from low-weight (LW), medium-weight (MW) and high-weight (HW) pig littermates; they each showed the different rate of proliferation. SCs from LW pigs have a significantly lower proliferation rate at day 3 compared with SCs from both MW and HW pigs [32]. In addition, the same results have been found in poultry. A previous study demonstrated that SCs taken out of breast muscle and leg muscle tissues from White Plymouth Rock and WENs Yellow-Feathered chicks (an indigenous Chinese breed) showed the different rate of proliferation at 72 h (unpublished data from Wang XQ et al.). *In vivo*, at any given time fewer SCs are available for fusion with existing muscle fibers because of the slower proliferation rate. When SCs are isolated from their natural environment and cultured *in vitro* under the same culture conditions, differences in the SC proliferation rate, as found in this study, must be due to the SCs themselves. Differences in the proliferation potential of SCs *in vivo* can be explained by differences in the nutrient supply, quantity of growth factors and fiber type. Thus, when SCs from the two breeds are cultured *in vitro*, differences in proliferation potential must be due to genetic factors. Our data indicated that significant differences in the proliferation of SCs in Lantang and Landrace pigs at 72 h may be an important point for further research.

A sustained proliferative response requires the coordination of both cell cycle progression and cell growth (increases in cell size and cell mass) [33], [34]. To remain constant during proliferative conditions, both DNA content and cell mass must double during the course of each cell division cycle. In this study, the SCs from Lantang and Landrace pigs proliferated to 48, 72, 96 and 120 h were tested for DNA content to count the distribution of cells in the G_0_/G_1_, S and G_2_/M phases of the cell cycle. Our results revealed that the percentage of cells in the G_0_/G_1_ phase for Landrace pigs was significantly higher, whereas of S and G_2_/M cells were significantly lower respectively than for the Lantang pigs at 72 h (Fig. 3 C–G), implying that the proliferation ability of SCs in Lantang pigs was higher. Furthermore, the mRNA levels of Myf5, MyoD, myogenin and Pax7 in SCs from Lantang pigs were significantly higher, while the transcript levels of myostatin and its downstream gene Smad3 were significantly lower than the Landrace pigs (Fig. 4 A). At the protein level, the MyoD and myogenin in Lantang pig SCs were significantly higher, whereas the myostatin was significantly lower than in Landrace pigs (Fig. 5 A–C). Previous studies have indicated that MRFs function in processing myogenesis, and their expression has been used as an indicator of muscle development [35], [36]. In quiescent SCs, Pax7 is expressed. After activated, Pax7 and MyoD are co-expressed [37]–[39]. Myostatin, also called growth differentiation factor-8, is a TGF-β family member that acts as a negative master regulator of skeletal muscle mass [40]–[42]. Exposure to myostatin induces cyclin D_1_ degradation and causes cell cycle arrest [40]. Our data are similar to previous studies. High expression of MyoD (mRNA and protein) increased the rate of proliferation in SCs from Lantang pigs. However, we found that the proliferative potential of SCs from Landrace pigs was negatively regulated by myostatin (mRNA and protein). According to Yang's study [40], we speculated that SCs from Landrace pigs may be negatively regulated by myostatin by inducing cyclin D_1_ degradation to cause cell cycle arrest. These findings were further supported in our cell proliferation study. Our results also suggested that the number of activated SCs (Pax7^+/^MyoD^+^) in Lantang pigs was greater than in Landrace pigs in proliferation at 72 h, and myostatin mediated its downstream signaling gene Smad3 to regulate SC proliferation. However, further studies are required to elucidate the precise molecular mechanism.

Interestingly, while examining the mRNA levels of mTOR pathway, we found that the transcript levels of Akt, TSC1, mTOR, eIF4E, S6K and RPS6 were significantly higher in SCs from Landrace pigs than in Lantang pigs (Fig. 4 B & C). The protein levels of S6K, phosphorylated mTOR and phosphorylated eIF4E in SCs in Landrace pigs were significantly higher than in Lantang pigs (Fig. 5 D–F). Current studies indicated that myostatin activity in mature muscle tissue is sufficient to inhibit the myofibrillar synthesis rate and phosphorylation of S6K [43], and its overexpression down-regulates Akt/mTOR signaling [41]. Furthermore, myostatin reduces Akt/TORC1/p70S6K signaling by inhibiting myoblast differentiation and myotube size [42]. In our study, the expression levels of myostatin, S6K, mTOR and eIF4E (both mRNA and protein) in SCs of Landrace pigs were significantly higher than concurrently in Lantang pigs, which contradicted previous studies by Welle [43], Amirouche [41] and Trendelenburg [42]. We elucidated that cell cycle progression and cell growth are separable and distinct [13]. Furthermore, mTOR controls mammalian cell size and cell cycle progression via its downstream targets p70S6K and 4EBP1/eIF4E [13], [14]. Although both cell size and cell cycle progression are controlled by mTOR and mTOR-dependent signaling pathways, the nutrient- and mitogen-responsive signaling molecule is centrally positioned to couple cell growth with cell division when the cells are cultured under the same conditions in which nutrients are restricted. With the evidence that cell cycle progression is dependent on a sufficient level of cell growth [44], [45], mTOR primarily drives cell growth (cell size) and as a secondary consequence, promotes cell cycle progression. *In vivo*, the fiber size in Landrace pigs was larger, whereas there were fewer fibers in Lantang pigs (Fig. 1 A–G). The SC cell size in Landrace pigs was also larger than in Lantang pigs *in vitro* (Fig. 3 H). Collectively, our findings suggested that high expression levels of Myf5, MyoD, myogenin and Pax7 increased the cell numbers in Lantang pigs, while myostatin negatively regulated the SCs in Landrace pigs. Furthermore, under the same nutrient-limited culture conditions, *in vitro* cell size is predominantly regulated by mTOR and its downstream targets S6K and eIF4E. However, further studies are required to confirm this molecular mechanism.

In conclusion, the proliferative ability of SCs seems to be dependent on the host from which they were collected. Our results suggested that the proliferative potential of SCs in Lantang pigs is higher than in Landrace pigs. The precise molecular mechanism that leads to this difference is currently unknown, but it is likely that it is a component of the myostatin and mTOR signaling pathways.
